# Probing the Dynamics of Identified Neurons with a Data-Driven Modeling Approach

**DOI:** 10.1371/journal.pone.0002627

**Published:** 2008-07-09

**Authors:** Thomas Nowotny, Rafael Levi, Allen I. Selverston

**Affiliations:** 1 Centre for Computational Neuroscience and Robotics, Department of Informatics, University of Sussex, Falmer, Brighton, United Kingdom; 2 Escuela Politechnica Superior, Universidad Autonoma de Madrid, Madrid, Spain; 3 Institute for Nonlinear Science, University of California San Diego, La Jolla, California, United States of America; University College London, United Kingdom

## Abstract

In controlling animal behavior the nervous system has to perform within the operational limits set by the requirements of each specific behavior. The implications for the corresponding range of suitable network, single neuron, and ion channel properties have remained elusive. In this article we approach the question of how well-constrained properties of neuronal systems may be on the neuronal level. We used large data sets of the activity of isolated invertebrate identified cells and built an accurate conductance-based model for this cell type using customized automated parameter estimation techniques. By direct inspection of the data we found that the variability of the neurons is larger when they are isolated from the circuit than when in the intact system. Furthermore, the responses of the neurons to perturbations appear to be more consistent than their autonomous behavior under stationary conditions. In the developed model, the constraints on different parameters that enforce appropriate model dynamics vary widely from some very tightly controlled parameters to others that are almost arbitrary. The model also allows predictions for the effect of blocking selected ionic currents and to prove that the origin of irregular dynamics in the neuron model is proper chaoticity and that this chaoticity is typical in an appropriate sense. Our results indicate that data driven models are useful tools for the in-depth analysis of neuronal dynamics. The better consistency of responses to perturbations, in the real neurons as well as in the model, suggests a paradigm shift away from measuring autonomous dynamics alone towards protocols of controlled perturbations. Our predictions for the impact of channel blockers on the neuronal dynamics and the proof of chaoticity underscore the wide scope of our approach.

## Introduction

To ensure survival in an unforgiving world, some essential motor patterns, e.g., for heartbeat, breathing or digestive movements, need to be sustained within tight operational limits. To what extent these limits on the functional output imply similar tight limits on the properties of the neuronal circuits is a challenging question because it entails many technical and conceptual difficulties. There are two extreme scenarios of how control of cellular properties may be accomplished. In one scenario the neuronal properties are fully genetically determined as in the notion of identified neurons [1]–[3]. In the other proposed scenario, the same operational limits may be achieved in many different ways, while allowing corresponding neurons in different individuals to be almost entirely different in their biophysical composition [4]–[6]. The biological reality for the control of cellular properties likely lies between these limit scenarios. It therefore seems advisable to approach this question with quantitative techniques that will help determine how the composition of the neural currents is produced and how it is maintained throughout protein turnover and changing conditions. Here, we aim to quantify how tightly cellular parameters may be controlled, by combining electrophysiology and detailed modeling of an identified neuron.

Due to its excellent experimental accessibility and stability, and the vast existing literature for many of its properties, the stomatogastric system of the lobster is well suited for approaching this question. It has been shown that the pyloric rhythm is very consistent from animal to animal [4], [7] during development of body size [8], under natural [9] and artificial perturbations [10], and even after decentralization [11]–[13]. Furthermore, the cells generating this rhythm exhibit stereotyped dynamics and can be identified by visual inspection of intracellular recordings and comparison to simultaneous extra-cellular recordings of identified nerves.

Neuronal circuits may be more variable in mammalian systems and the answers to our questions of how robust target dynamics are achieved are likely to vary among different systems depending on their function and their ensuing accuracy requirements. The heartbeat, for example, may be more tightly controlled than digestive movements. However, mammalian neurons can often also be clearly characterized by their stereotyped dynamical properties [14] and genetic signature [15] and in many respects questions pertaining to cortical function can be analogous to similar questions in simpler pattern generators [16] like the pyloric system. Furthermore, while the specific detailed requirements of different systems may be different, it is likely that general principles exist that govern the way unlearned rhythmic behaviors are produced. It is these principles that we are trying to uncover.

We approached the question of which neuronal properties are controlled (and therefore most consistent across preparations) by analyzing the dynamics of isolated lateral pyloric (LP) neurons of the lobster. Like other neurons in this network, the LP is a conditional burster, requiring modulatory input from higher centers to burst [17]. Its biophysical and dynamical properties have been studied extensively [18]–[21]. In our analysis we followed a two-pronged approach: First, we acquired data from isolated LP neurons in many different conditions and analyzed the observed dynamics directly, continuing earlier work on LP [22].

We then used the extensive data obtained in the first phase to build an accurate conductance based model of the LP neuron which we explored numerically. The structure of conductance based neuron models is motivated by the biophysics underlying membrane potential generation by ionic channels. Using this type of model should, therefore, allow us to predict neuron behavior beyond a mere mimicry of the target data used. This predictive power has made conductance based neuron models the gold standard for data-driven model development in computational neuroscience and recommended them for our purposes.

Conductance based models are notoriously hard to adjust to observed data. Therefore, we chose to use automated fitting (parameter estimation) techniques both for finding the kinetic parameters of conductances from sets of voltage clamp data [18], [19], [23], [24] and for estimating parameters of the model as a whole when aiming to replicate our own large sets of current clamp data.

In earlier comparative work on parameter search methods for H-H neuron models [25], good performance was found for genetic algorithms [26] and simulated annealing [27], [28] and no significant performance differences between these two types of optimization algorithm was observed. In the work presented here, we chose to use simulated annealing over genetic algorithms guided by the long-term perspective of using the developed parameter estimation technology online in the future.

Models of the LP neuron of the lobster and crab have a long tradition and were developed in roughly three independent strains. One strain is based on voltage clamp recordings of identified isolated LP cells in the crab [18], [19], [23] which were assembled into an early LP neuron model [24]. This model was used with several variations in subsequent work [21], [29], [30]. A second strain of models was based on voltage clamp data from unidentified cultured stomatogastric neurons of the lobster [31] which were, with modifications, assembled into an LP neuron model [32] that became the basis of several later studies as well [33]–[37]. The later models differed from the original model [32] mainly by changes in the *I_h_* current. The third group of models were more abstract models based on the Hindmarsh-Rose equations [20], [38]–[40].

All existing models were successful in illustrating different aspects of CPG function while (necessarily) neglecting others. In particular, predicting the effect of perturbations on the dynamics of isolated LP neurons proved difficult with the existing models. The model developed here builds on the existing models and aims at extending them to allow accurate model behavior over a wider range of conditions. The question of how much this increased range of accuracy improves the predictive power of the model for increasingly severe perturbations will be addressed in the sections on parameter sensitivity below.

In the remainder of this work we also analyze the origin of the irregular dynamics observed in the LP membrane potential over a wide range of conditions. To this end we are using our model as a proxy system to learn about the neurons it describes. This approach allowed us higher mathematical confidence in our numerical results than the direct non-linear time series analysis used previously. We find that the model is chaotic over a wide range of conditions and that the chaoticity typically persists even if parameters of the model are changed.

## Results

To characterize the difference among identified neurons of the same type we isolated 6 LP neurons from the lobster stomatogastric ganglion and compared their activity. We inspected a wide dynamic range of the neurons activity by injecting DC currents ranging from −4 to +2 nA, a range that is likely to encompass the inputs that LP neurons may encounter when in the intact network.

### Properties of isolated LPs

**Figure 1:**
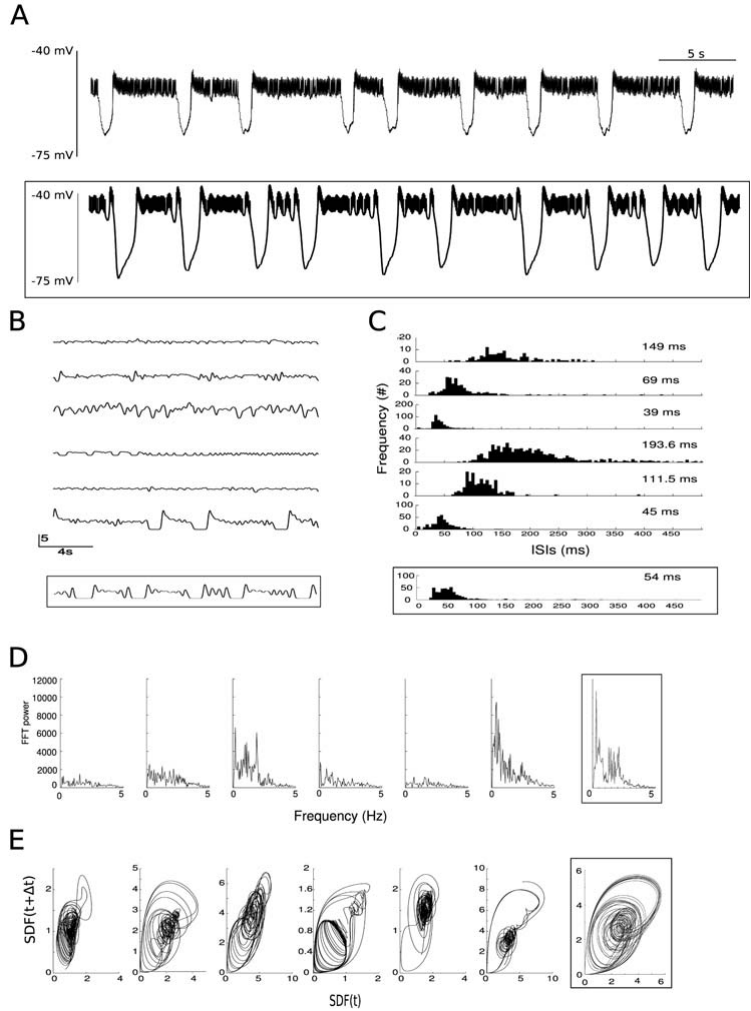
Properties of isolated LP neurons without current injections. The framed panels show data from the LP model for comparison. A) Example of the membrane potential time series of an isolated LP neuron. Irregular spiking is interspersed with occasional hyperpolarization events. B) The SDFs of six isolated LPs show a quite variable range of activities. The sixth data set, here and in the following panels, was the one used for the model fitting. C) Histograms of the inter-spike intervals for the different LP neurons. The median for each distribution is given on the top right of the panels. D) The frequency spectrum of the LPs as calculated with an FFT of the SDFs shows a wide distribution of frequencies without marked peaks in all LPs. This is indicative of irregular spiking neurons. E) SDF return maps of the isolated LPs with embedding delay Δ*t* = 100 *ms*. The condensed areas are the irregular spiking regions and the larger loops the hyperpolarization events followed by the high frequency burst onset.

When the LP is embedded in the pyloric circuit, its burst durations, burst frequency and duty cycles are quite consistent from one preparation to the next [7], [8], [10]. It is, however, not clear how much of this consistency is due to a corresponding consistency in the cellular properties, how much is due to properties of the synaptic connections or general network connectivity, how much is due to cellular- and network-level regulatory mechanisms and how much is due to non-linear dynamical interactions between the neurons in the network. To approach these questions, we sought to investigate the properties of LP neurons that have been isolated from the rest of the network by blocking all synaptic glutamatergic inputs into the neuron with a pharmacological blocker (PTX) and by photoablation of other neurons (see Methods). We analyzed the isolated LP neurons from 6 different animals. Following isolation the typical activity of the LP neuron is irregular spiking with occasional hyperpolarization as shown in the representative example in Figure 1A (see also [39]). The intervals between hyperpolarization events vary as well as their amplitudes, the former effect also clearly visible in the traces of spike density functions (SDFs) for the different LPs (Fig. 1B). The inter-spike interval (ISI) distributions shown in Figure 1C reveal an approximately three-fold variation in the mean ISI (see Table 1). The long tail on the right of the histograms contains the intervals that correspond to hyperpolarization events. The spike timing characteristics of all observed neurons were typical for irregularly firing neurons with wide power spectra that are lacking clear peaks (Fig. 1D). The evolution of spiking activity as illustrated by the return map (Fig. 1E) shows rather different durations of hyperpolarization events and maximal as well as typical spike densities for individual neurons. The distributions of ISIs show similar variability in their skewness and kurtosis (Table 1). The implications for the reproducibility of properties of LP neuron dynamics will be discussed in more detail below.

**Table 1:** Dynamical properties of isolated LP neurons in control conditions.

Experiment	Mean	SD	Skewness	Kurtosis
1	160.0	50.4	0.89	3.5
2	81.5	50.2	3.70	21.4
3	46.6	35.7	5.89	46.4
4	204.3	76.2	1.43	5.1
5	115.7	33.7	3.63	28.3
6	46.7	20.8	1.16	7.1

The distributions of inter-spike intervals of different LP neurons differ in terms of mean and standard deviation (also compare to figure 1C) on a similar order of magnitude as on higher order statistics like the skewness and kurtosis. The skewness is, however, always positive which is consistent with a typical burst shape in the LP neuron [46].

Under natural conditions, LP cells are embedded in a network and constantly receive many, mainly inhibitory, inputs. The condition of complete autonomous dynamics in isolation is accordingly rather unnatural for these cells. Therefore, it is actually more relevant to examine them under the influence of current injections. From a dynamical systems point of view it appears equally important to probe the neurons, seen as dynamical systems, in a large area of the state space to gain a meaningful overview over their (dis)similarities. Here we started with a simple protocol of constant DC current injections that roughly mimics the slow inhibitory currents and release from such currents encountered by the LP neurons through graded inhibitory synapses [17], [41] in their natural mode of operation.

### Dynamical Properties of isolated LPs

**Figure 2:**
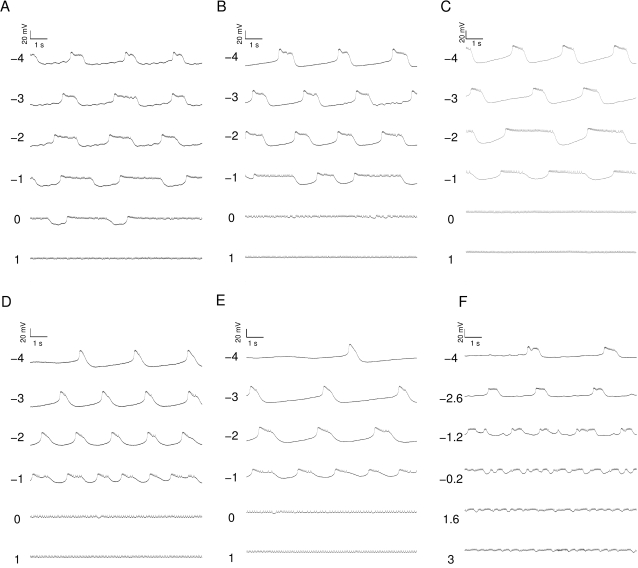
Membrane potential of six isolated LP neurons subject to current injection of −4, −3, −2, −1, 0, and 1 *nA* (A–E) and −4, −2.6, −1.2, 0.2, 1.6, and 3 *nA* (F). The specific activity of neurons at given injection levels varies considerably. The overall transition from short bursts to increasingly longer and less regular bursts to irregular and eventually tonic spiking is, however, well preserved. The data used for model development is shown in panel A.

**Figure 3:**
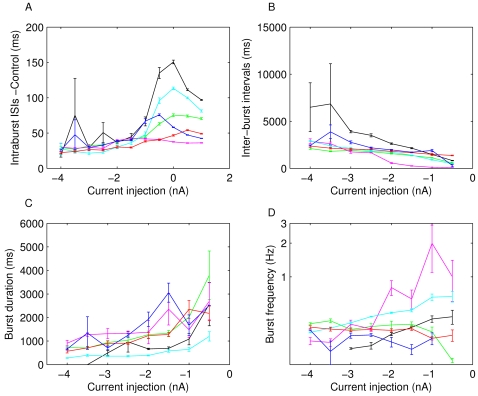
Dynamical properties of LP neurons from 6 lobster preparations. All properties are measured during 20 s constant DC current injections *I_DC_* of different levels (x axes). A) Average ISI of spikes within bursts, B) intervals between the end of a burst to the beginning of the next burst (inter-burst interval, IBI), C) Burst duration and D) burst frequency as measured from burst onset to burst onset. All properties exhibit a fair amount of variability between preparations while the general trend of the dependence on current injection is better conserved. The intra-burst ISI increases with *I_DC_* up to *I_DC_* = 0 *nA*, then decreases, the IBI decreases with increasing *I_DC_*, and the burst duration increases with increasing *I_DC_*. The burst frequency does not show a very clear trend.

As much as the neurons differ while in isolation, and possible reasons for that will be discussed below, once studied with DC current injections, the neurons reveal more consistent features. For instance, the dynamical profile of the different LPs is rather similar (Fig. 2). It manifests itself primarily in a typical transition from regular bursting with short bursts when strongly hyperpolarized to progressively longer bursts then irregular bursting and eventually tonic spiking. A quantification of these observations is shown in Figure 3. The average inter-spike interval (ISI) within bursts (Fig. 3A) gradually increases with increasing *I_DC_* in the deeply to moderately hyperpolarized regime (−4 to −1 *nA*) it levels out and starts decreasing for positive current injections, see also [22]. The inter-burst interval (IBI) shown in Figure 3B (only analyzed in the range of current injections where clear bursting was present) has a clear decline for increasing *I_DC_* while the burst duration always increased with decreasing hyperpolarization (Fig. 3C). Finally, the burst frequency (Fig. 3D) increased as the membrane potential was increasingly less hyperpolarized in three out of the six experiments. For two others it didn't change significantly and for one it decreased. This mixed result makes the change in burst frequency as a function of current injection the least consistent of the observed trends even though we would like to argue that some tendency to increase on average seems to exist. A summary of all observed correlations and the corresponding P values is shown in Table 2.

**Table 2:** Correlation of dynamical properties with current injection.

Experiment	Intraburst	Interburst	Burst	Burst
	Intervals (R, P)	intervals (R, P)	duration (R, P)	frequency (R, P)
1	0.7522, 0.0076	−0.9593, <0.001	0.8484, 0.0078	0.9876, <0.001
2	0.9397, <0.001	−0.9413, <0.001	0.8375, 0.0095	−0.7038, 0.0514
3	0.6102, 0.0462	−0.9657, <0.001	0.8041, 0.0161	0.7510, 0.0317
4	0.8572, <0.001	−0.9898, <0.001	0.8193, 0.0128	0.9721, <0.001
5	0.9572, <0.001	−0.9924, <0.001	0.9228, 0.0011	−0.7779, 0.0230
6	0.5450, 0.0829	−0.8114, 0.0145	0.7781, 0.0230	−0.3149, 0.4915

Pearson's R correlation of the dynamical properties with the current injection level. The correlation of each of the variables in Figure 3 with the current injection level was calculated (R) and is tabulated with the P value for testing the hypothesis of no correlation against the alternative that there is a non-zero correlation.

Even though the specific values of the properties are different among the different LPs, the changes of these variables in response to different current injections are fairly consistent. This consistency of changes, or “trends”, rather than absolute values, raises the question of what kind of restrictions may be imposed on which properties of the LP neurons and how tightly these restrictions may have to be to allow for consistency of trends. We chose to explore this question by studying a data-driven model of the isolated LP neuron.

### Model parameter estimation

Rather than building a model specifically designed to shed light on the question of preserved trends in neuron properties we aimed at building a general conductance-based model of the LP neuron that replicates LP neuron dynamics over a wide range of conditions. In particular, to avoid bias, the above-mentioned consistent trends were not used as a selection criterion during the model development.

Previous reports [18], [19], [24], [31], [32] have focused on the match of models to voltage clamp data and current clamp data in control conditions. In contrast, we required that the response to DC current injections, as well as the transient behaviors when current is introduced and when the cell is released from injections, are matched by the model. The extent to which this requirement restricts the model sufficiently to allow the prediction of, e.g., the action of ion channel blockers, is not obvious and, therefore, part of our investigation.

In preliminary studies we tested different types of data sets and eventually selected a long, uninterrupted recording of the membrane potential of an isolated LP cell in response to current steps of 20 s duration and equidistant levels from −4 *nA* to +1 *nA* in steps of 0.5 *nA*, which is a realistic physiological range for the LP cell. LP cells typically become silent at stronger hyperpolarizations and suffer depolarization block and potentially cell damage for stronger depolarizations. The current steps were separated by 20 s of autonomous behavior without current injection. The 20 s time scale of steps was chosen based on the typically observed duration of transient behavior of LP in response to current steps which were on the order of 10 s.

In automatic parameter estimation (fitting) procedures the quality of any given model, i.e., a given set of parameters, is assessed by a so-called cost function. The choice of the appropriate cost function is one of the major factors in determining the success of the parameter estimation. For building our conductance based model of the LP cell we developed a cost function consisting of three main components (see the Methods): (i) The Euclidean distance of normalized SDF waveforms, (ii) the absolute difference of the integrals of the SDFs, and (iii) the Euclidean distance of the moving average of the membrane potentials. The three components were evaluated over 40 s time steps and then combined in a weighted sum to an overall performance measure (cost function). The weights were chosen by manual inspection such that the normalized SDF contributed typically approximately 90%, the integral of the SDF about 9% and the moving average of the membrane potential about 1% to the overall cost. These contributions reflect our initial hypothesis of which dynamical properties may matter most for the correct function of the LP neuron in the circuit: The pattern of bursting, measured by the normalized SDF, is functionally most important. Only if this is almost equally well reproduced by two models, we consider the overall spike rate, i.e., the spiking frequency within bursts, as a secondary criterion - hence the 10 fold less weight for the integral of the SDF. If this is also equally well-reproduced (another 10 fold reduction of weight) we would choose the model whose membrane potential waveform, reflected by the moving average of the membrane potential, is closer to the observed data.

During the parameter estimation procedure the model is compared to the data simultaneously in 10 sliding windows of 40 s width. The composite cost function values from all windows are summed before examination by the optimization algorithm thus allowing trade-offs between improvement in some of the windows and deterioration in others. As each time window contains exactly 20 s of data with current injection and 20 s of data in control conditions, these trade-offs correspond to trade-offs between different current injection levels .

Due to the fact that the model was not compared to the full data set during each annealing step, there is no measure of absolute cost or performance. To assess whether suggested parameter changes improved model performance we compared a model with modified parameters to a model with the original parameter set, but otherwise in the exact same conditions.

In each given step, parameters were randomly chosen to be adjusted with probability *p_perturb_*. The size of suggested parameter changes was adjusted according to the observed sensitivity of the model to changes in the parameters at any given time and the total range of parameter values was hard-limited to specific intervals (see Methods).

In the final form of the algorithm we adjusted 20 parameters (see Table 3, which also contains the final parameter values after optimization).

**Table 3:** Parameter values that were adjusted during the parameter estimation procedure and their final values.

Conductances	*g_CaT_*	*g_CaS_*	*g_KCa_*	*g_A_*	*g_h_*	*g_leak,a_*	*g_leak,s_*
	11.23 µS	6.428 µS	149.7 µS	72.08 µS	1.142 µS	0.1004 µS	0.06211 µS
Other	*C_a_*	*C_ICa_*	*k_Ca_*	*V_shift_*	*g_w_*	*C_s_*	*I_scale_*
	11.48 nF	505 M/As	17.3 Hz	−9.343 mV	0.4017 µS	5.439 nF	1.223
M current	*g_M_*	*V_mM_*	*s_mM_*	*k_mM_*	*V_kmM_*	*s_kmM_*	
	26.13 µS	−26.99 mV	−5.957 mV	0.1387 Hz	−60.58 mV	−13.26 mV	

The activity of isolated LP neurons is highly irregular in a wide range of conditions. The main difficulty in using such irregular (potentially chaotic) activity patterns for parameter estimation is the problem of proper alignment, which is aggravated by the nature of spikes in neuronal data. Our smoother cost function already partially addresses this problem. In addition we built on earlier work on similar problems [42]–[44] and introduced a simulated electrical coupling between the data and the model, see also [45]. This coupling term enforces a certain degree of synchronization of the slow dynamics allowing for a more objective assessment of model performance. During the parameter estimation procedure, the coupling strength is then systematically reduced.

**Figure 4:**
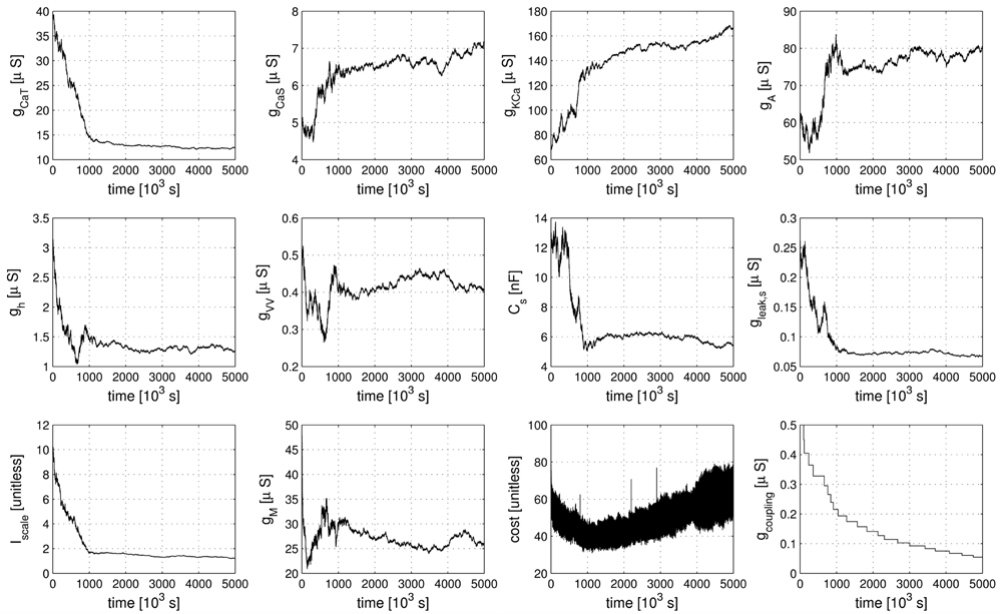
Evolution of 10 of the 20 estimated parameters (see text) during the fitting procedure. Parameters tend to converge to a final value aided by a slow decrease of the size of explored parameter changes over the extent of the whole fitting procedure. The two bottom right panels show the composite cost function after each accepted parameter change (second to last panel) and the strength of the simulated electrical coupling (last panel, see Methods). The strong oscillations in the cost function are due to the model being compared to a different partial data set at each step of the parameter estimation procedure. The increase of the cost function in the late stages of the procedure are due to reductions in coupling strength which can only be partially compensated by the subsequent compensatory parameter changes and does not indicate a lack in convergence. In a sense, the stronger coupling earlier in the procedure feigns error function values that are lower than what can actually be achieved at lower coupling. The data displayed encompasses 4·10^5^ simulated seconds and was produced in approximately 100 CPU days on an AMD Athlon 2200+ based platform.

The evolution of parameter values during the fitting procedure is illustrated in Figure 4. All parameters eventually converged to stable values helped by the continuous gradual reduction of annealing temperature and target lateral cost, which was governing the parameter-change step sizes (see Methods). The simultaneous stepwise reduction of the coupling strength between data and model has the somewhat opposite effect of encouraging more changes in parameters because reducing the coupling strength induces appropriate compensatory parameter adjustments. It is noteworthy that the cost function increased slightly in the later stages of the procedure seemingly indicating that the procedure did not converge properly. This is, however, again due to the decreasing coupling strength which induces higher cost function values that in turn are only partially compensated by the subsequent parameter changes. The fitting procedure was terminated when the coupling reached small values in physiological terms and the parameters became stable.

By visual inspection, the final model exhibits dynamics very similar to the dynamics of LP neurons (Fig, 1A, 2A compared to 5A). For a realistic comparison to *in vitro* electrophysiological recordings we added weak low-frequency noise to the LP neuron model (see Methods for details) in all analyses except for the calculation of Lyapunov exponents. Visible deviations from the LP data used for developing the model seem well within the range of the observed variability between different LP neurons (compare to Fig. 2).

**Figure 5:**
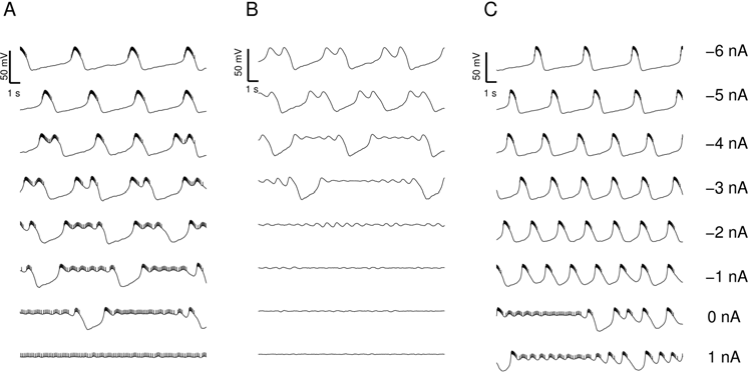
Predictions of the model for blocking selected currents. A) Model membrane potential time series in control conditions. B) With the sodium conductance blocked, the membrane potential of the model continues to oscillate in ‘spike-less bursts’ in analogy with earlier observations in the pyloric system [49] C) When the A current is blocked (*g_A_* = 0) the model continues almost normal activity with slight changes in the burst frequencies and the locus of transition from bursting to spiking.

### Dynamical properties of the LP model

As shown in Figure 1, the direct membrane potential, the histogram of ISIs, the power spectrum and the SDF return map of the model are within the range of those observed in biological LPs. Note, however, the slight differences in the actual amplitude and width of the spikes as seen in the soma compartment (compare Fig. 1A). This effect is not unexpected because the membrane potential does not directly enter the cost function in the parameter estimation procedure. The relative similarity of the spiking-bursting waveform of the membrane potential to the experimental observations is on the contrary, rather surprising considering this lack of constraint by the cost function.

**Figure 6:**
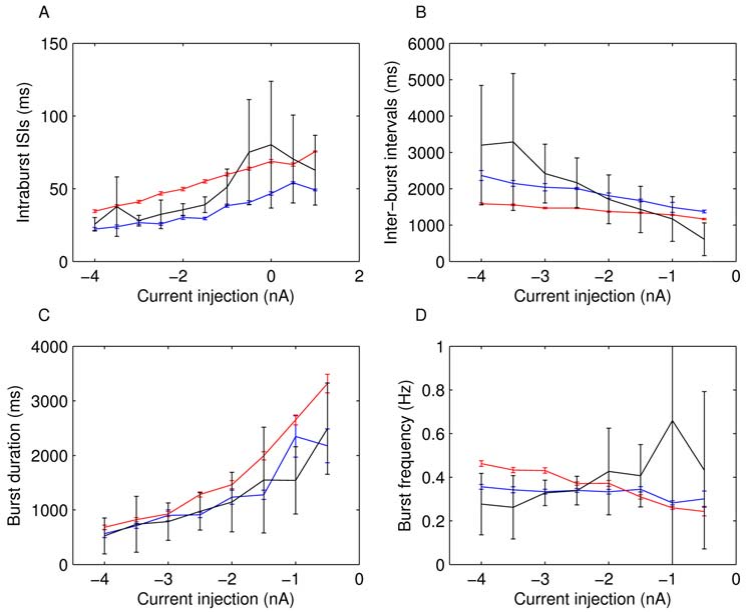
Intraburst ISI (A), inter-burst intervals (B), burst durations (C) and bursting frequency (D) of the fitted LP (blue), the model (red) and the average of all measured LP neurons (black) in comparison. The error bars mark the standard error. The model and the experiment correspond very well with the exception of slightly less fast spiking within bursts in the model. For most current injections the model properties also fall well within the range of data observed in the different experiments.

Encouraged by the positive outcome of the basic dynamical profile of the model we then turned to our question of specific trends in the properties of neuron dynamics. Figure 6 compares the dynamical properties of the fitted model to the properties observed in the LP neuron whose data was used in the fit. All trends are reproduced and the burst duration and -frequency are even quantitatively close to those of the biological neuron.

**Figure 7:**
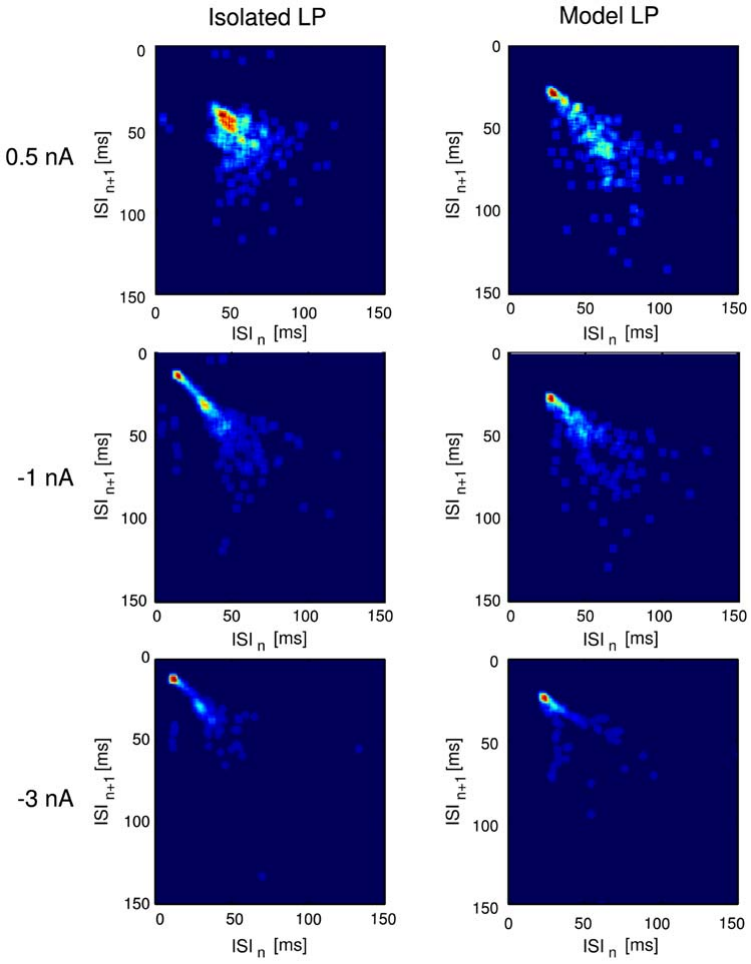
Return maps of ISIs in the biological LP and the model LP at three levels of current injection (indicated on the left of the panels). The characteristic V-shape structure of the LP neuron [46] is well reproduced.

A more rigorous test of the fitting are the ISI return maps of the biological LP and the model LP at three different current injection levels, shown in Figure 7. The figure shows the dependence of each ISI on its preceding spike and the map's structure is indicative of the underlying bursting-spiking dynamics. During tonic spiking the shape of the map is a triangular cloud (Fig. 7A) that turns into a V-shape structure in the bursting regime at larger hyperpolarizations. This V-shape map indicates that a portion of short ISIs are followed by longer ISIs and vice versa [46]. The reproduction of correct ISI return maps was not required from the model and only examined after the model was completely developed. Apparently, the fitting procedure in combination with the constraints posed by the overall structure of a conductance-based model with experiment based current kinetics were sufficient to enforce this neuron “signature” [46].

We have reproduced the general dynamical properties and the characteristic changes of dynamical properties in response to current injections in our model. We can now address our original questions of how sensitive the model, and, therefore, presumably the neurons, are to changes in their parameters. For clarity, we will focus the following discussion on maximal conductances of ionic currents even though the analysis was performed for all 69 parameters listed in Tables 3 and 4.

**Table 4:** Parameters that were not subject to fitting during the main parameter estimation procedure.

*I_CaT_*	*V_mCaT_*	*s_mCaT_*	*V_hCaT_*	*S_hCaT_*	*k_mCaT_*	*k_hCaT_*	*V_khCaT_*	*s_khCaT_*
	15 mV	−9.8 mV	−40 mV	3.2 mV	45 Hz	20 Hz	−15 mV	−10 mV
*I_CaS_*	*V_mCaS_*	*s_mCaS_*	*k_mCaS_*					
	29.13 mV	−4.431 mV	60.86 Hz					
*I_KCa_*	*c_mKCa_*	*V_mKCa_*_1_	*s_mKCa_*_1_	*V_mKCa_*_2_	*s_mKCa_*_2_	*c_hKCa_*_1_	*c_hKCa_*_2_	*kmKCa*
	2.5 µM	0 mV	−23 mV	−16 mV	−5 mV	0.7 µM	0.6 µM	600 Hz
	*k_hKCa_*	*f*						
	35 Hz	0.6 mV/µM						
*I_A_*	*V_mA_*	*s_mA_*	*V_hA_*	*s_hA_*	*k_mA_*	*k_hA_*_1_	*k_hA_*_2_	*V_kha_*_2_
	−12 mV	−26 mV	−62 mV	6 mV	140 Hz	50 Hz	3.6 Hz	−40 mV
	*s_khA_*_2_	*V_aA_*	*s_aA_*					
	−12 mV	7 mV	−15 mV					
*I_h_*	*V_mh_*	*s_mh_*	*V_kmh_*	*s_kmh_*	*k_mh_*			
	−70 mV	8 mV	−110 mV	−21.6 mV	0.33 Hz			
*V_x_*	*V_Na_*	*V_Kd_*	*V_KCa_*	*V_A_*	*V_h_*	*V_M_*	*V_leak_*	
	50 mV	−72 mV	−72 mV	−72 mV	−20 mV	−80 mV	−50 mV	
other	RT/F	*g_Na_*	*g_Kd_*	*P_Ca_*	[*Ca*]*_out_*	[*Ca*]_0_		
	11.49 mV	715 µS	143 µS	1.56 1/mM	15120 µM	0.02 µM		

The values for the parameters that were not subject to fitting in the main procedure were taken from the literature or from separate fits to voltage clamp data taken from the literature, see Methods.

### Sensitivity in terms of the cost function

Our first test of sensitivity was to determine the effect of parameter changes on the model dynamics quantified in terms of our cost function. We changed the parameters of the model one at a time and, using our cost function, compared the model output with the changed parameter to the original model, over 10 s time windows. The parameters were changed on a logarithmic scale to ensure fine initial stepping and, at the same time, sufficient coverage of potentially very large parameter ranges. Each trial was terminated if either the cost function (same as in the fitting procedure; see Methods) exceeded 100 or the parameter was changed 10-fold (multiplicative parameters) or by ±50 *mV* (additive voltage parameters).

For sufficiently small steps, the cost function grows approximately linearly with the size of the parameter change. We, therefore, fitted the cost as a linear function of the parameter change in % (multiplicative parameters), or mV (additive voltage parameters). We added points to the fit incrementally until the standard error of the linear regression reached a maximum of 0.1.

**Figure 8:**
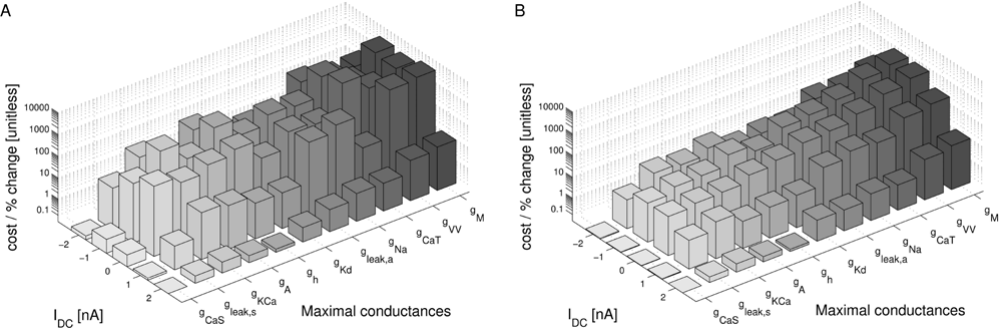
Sensitivity of the model to changes in conductance parameters in terms of the composite cost function. The bars (z axis) mark the rate of increase in the “lateral cost function” between the original model and a model with an incrementally changed parameter (x axis) at various levels of hyperpolarization (y axis). A) cost function increase rate for two uncoupled models measured over 10 s. B) cost function increase rate between models that were coupled with a one-directional ohmic coupling of 100 *nS* (analogous to the situation during fitting to experimental data). The conductances were ordered such that the *I_DC_* = 0 *nA* row in B) is in increasing order. For each parameter, the sensitivity to parameter changes is larger for intermediate *I_DC_* values and lower for strong de- or hyperpolarization. Note the logarithmic scale on the z axis implicating that the changes in sensitivity are quite drastic. Observed sensitivity ranged from 2.949·10^−4^/% to 4213/% in (A) and 1.712·10^−4^/% to 745/% in (B). The coupling not only considerably reduces the apparent sensitivity, but also makes the measurement more consistent across current injection levels: Unlike in (A) the order of sensitivities in (B) remains almost the same for the different current injection levels *I_DC_*.

The resulting parameter sensitivities of the model expressed in terms of the slope of the linear regression are shown in Figure 8A for 5 different hyperpolarization levels of the model neuron. As expected, different parameters can have very different effects. For instance, the parameter *g_CaS_* has very little influence on the measured output (order of magnitude lower than the next more important one) while the three most influential parameters are *g_M_* (spike rate adaptation in the axon compartment), *g_VV_* (coupling between compartments) and *g_CaT_* (transient calcium current). Not surprisingly, these parameters all play an important role in shaping the bursting dynamics, the two former regulating the influence of spikes on the burst termination, and the latter shaping the overall slow dynamics of the soma compartment that seems to be responsible for bursting (see Fig. 5B and the discussion relating to this below). There also are quite large differences of the effects of changing individual parameters at different DC current levels. Generally, the sensitivity to parameter changes is small for high depolarization and hyperpolarization levels, while it is large at intermediate levels of current injection. An exception to this rule are the leak conductance of the axon *g_leak,a_* and the sodium conductance *g_Na_* which have a flatter sensitivity profile with respect to current injections.

One may argue that the large cost function for some of the parameters and the corresponding large sensitivity to these parameters may be due to changes in timing and ensuing alignment problems as we discussed for the fitting procedure above. To address this problem we repeated the analysis with a simulated one-directional electrical coupling (*g_coupl_* = 100 *nS*, from the fixed model to the model with changed parameter), similar to the procedure of the model fitting. If the difference between the models is not too big, the coupling aligns the two models' slow bursting dynamics. This mechanism ensures that models that are very similar are not costed as being extremely dis-similar just because their bursts do not align. The corresponding sensitivity results are shown in Figure 8B. Comparing the uncoupled and coupled results, we notice that introducing the coupling not surprisingly reduced the overall sensitivity: the maximal sensitivity was 4213 per percentage change (4213/%) without coupling and 745/% with coupling (for *g_M_* in both cases); the minimal sensitivity was 2.949·10^−4^/% without and 1.712·10^−4^/% with coupling (for *g_CaS_*). Also, the sensitivity became more consistent across depolarization and hyperpolarization levels such that sorting by increasing parameter sensitivity at a current injection level of 0 nA causes all other levels to be almost perfectly ordered as well (this same order was used in both panels of Figure 8). Both changes indicate that some of the observed large sensitivity in the uncoupled case was indeed due to high cost functions induced by mis-alignment of the slow dynamics of the perturbed and unperturbed models. From an electrophysiological point of view the results obtained with the coupling are, therefore, probably more relevant and realistic.

### Tolerance level in terms of the cost function

**Figure 9:**
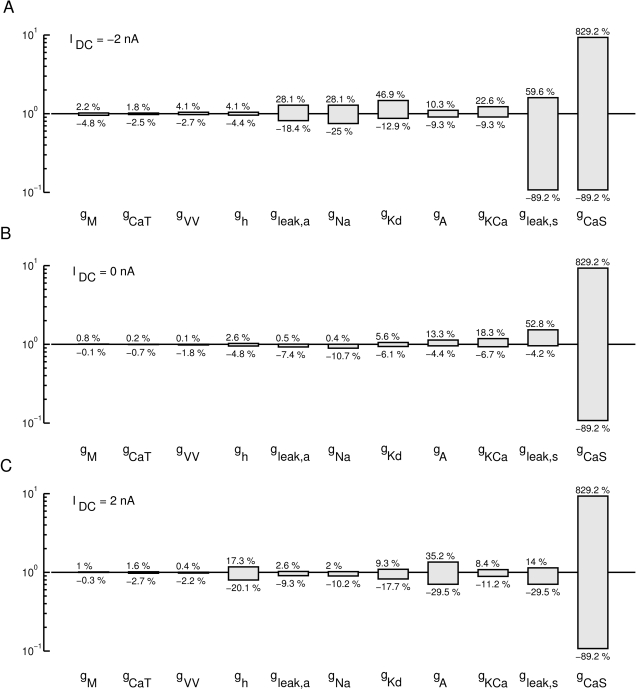
Tolerance regions for parameters based on the comparison of the model to itself using the composite cost function. Each bar marks the range of parameter change for which the “lateral cost function” between a perturbed and an unchanged model did not exceed 70, a typical value of cost between model and data at the end of the fitting procedure. Parameters were tested up to a 10 fold increase and 10 fold decrease. The parameters are ordered in increasing tolerance for *I_DC_* = 0 *nA* (B). Tolerances for some parameters are clearly increased for *I_DC_* = −2 *nA* (A) and *I_DC_* = 2 *nA* (C). Overall tolerance ranged from about 0.1% (e.g., *g_CaT_*) change to 900% change (*g_CaS_*). Most parameters are well-constrained with allowed tolerances of a few percent.

A different way of looking at the sensitivity to parameter changes is to look for the maximal interval around the “right” parameter value for which the cost function does not exceed a predefined threshold value. For our analysis we selected the approximate final average cost value of the fit to the data as a threshold, which was 70. The results are shown in Figure 9 for three different DC current injections, −2 *nA* (A), 0 *nA* (B), and +2 *nA* (C). Overall, the tolerance intervals for individual parameters vary considerably from [−0.1%, 0.8%] for *g_M_* at 0 nA current injection to no detectable restriction (tested up to 1/10 and 10 times original value) for *g_CaS_* in all conditions. The conductances are ordered according to the width of their tolerance interval at 0 nA current injection in ascending order from the left to the right. As in the sensitivity analysis above, *g_M_*, *g_CaT_*, and *g_VV_* are the most important parameters, i.e., need to be controlled most tightly, and *g_CaS_* is the least important.

The parameter *g_VV_* of coupling between compartments describes the interaction of currents by passive electrical conductance along the neuropil. The observed high importance of this parameter in the model indicates that the fact that the currents are not all co-located in one electrical compartment - an experimental fact indicated by the strongly attenuated spikes - is relevant for the neuronal dynamics.

Contrary to common intuition, we find the Ca induced potassium current, *g_KCa_*, and the A current, *g_A_*, in the range of low sensitivity as well. We have to keep in mind that the measure of sensitivity employed so far is determined by the choice of the cost function. We will see below that the apparent low importance of *g_KCa_* and *g_A_* will change if we consider the above-mentioned more robust trends in the LP neuron dynamics.

### Sensitivity in terms of general dynamical trends

As we described above, the LP neuron dynamics are variable across different animals while preserving some general statistical properties or more precisely, trends in such properties. In particular, the trend of how intra-burst ISIs, inter-burst intervals, burst duration, and, to lesser extent, burst frequency change as a function of DC current injection seems conserved across all analyzed LP neurons. One possibility may, therefore, be that this trend is what needs to be tightly controlled rather than the specific dynamics, a requirement that led to the very high sensitivities to some parameter changes in the previous section. To follow this idea, we systematically generated LP neuron model data for DC current injections from −4 to −0.5 *nA* in steps of 0.5 nA and with the usual, weak low-frequency noise. This data was obtained for the original model and for models in which one parameter at a time was changed in increasing steps (see Methods for details). For each parameter value we performed a linear regression for the 4 characteristics as a function of the hyperpolarization level. We continued changing a given parameter until one of the following conditions was fulfilled: (i) The characteristic trend had changed sign, (ii) the linear regression had an error greater than the absolute value of the parameter, or (iii) the hard upper or lower limit for the parameter value (10 fold change and 1/10 of the original value, respectively) was reached.

**Figure 10:**
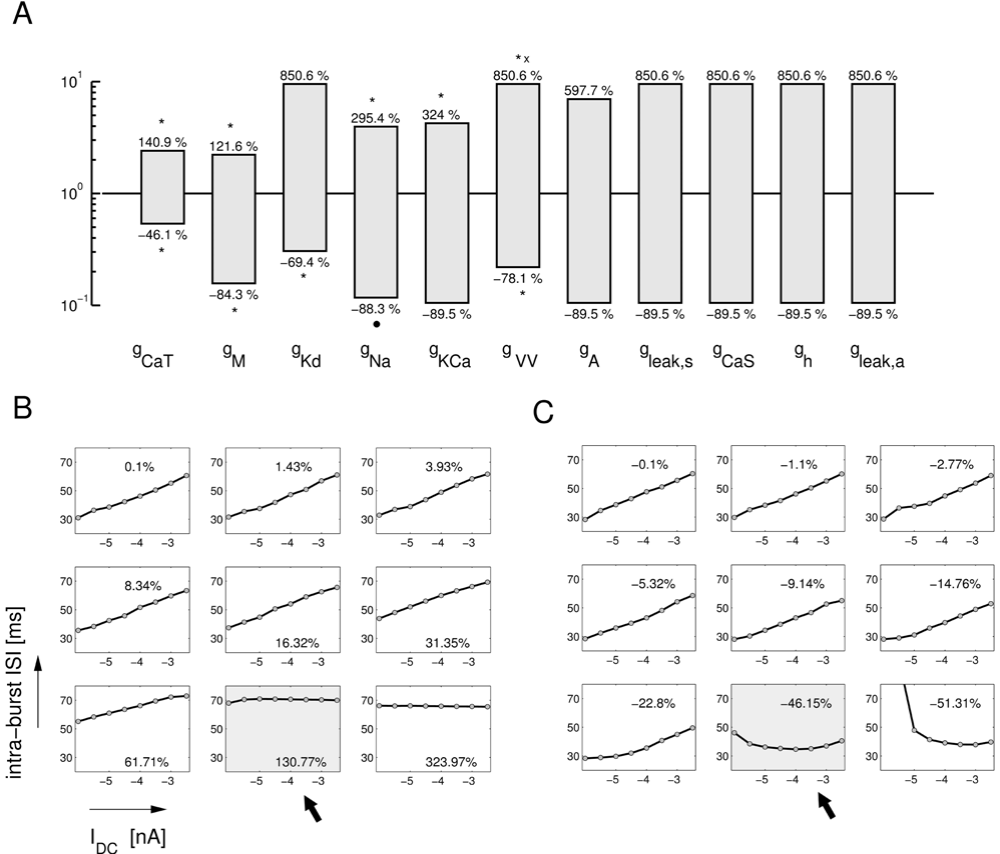
Model tolerance against parameter changes with respect to general trends in intra-burst ISI as a function of the current injection *I_DC_*. (A) The bars show parameter ranges in which the trend of intra-burst ISI as a function of the current injection *I_DC_* is preserved. (B) Examples for increases of the *g_CaT_* parameter. Each small panel shows the intra-burst ISI as a function of *I_DC_* for a given change of *g_CaT_* (in % of the control value) as noted within the panels. The trend of the intra-burst ISI remains monotonically increasing until a +130.77% change in *g_CaT_* (arrow). (C) Examples for decreases of *g_CaT_*. Here the trend changes for a 46.15% decrease of *g_CaT_*. The size of the bars in (A) was determined by the % change in each parameter for which the trend changed (like in examples B and C; marked with an asterisk), for which the trend is lost (the linear regression used to detect a trend has a large error; marked with an ‘x’), or for which the maximal change of an approximately 10 fold increase or decrease is reached (no mark). The lower bound on *g_Na_* marked by the bullet was defined by the complete cessation of spiking altogether. The large tolerance intervals found demonstrate that most parameters do not change or destroy the intra-burst ISI trend. The model is remarkably robust with respect to this general property.

Figure 10 illustrates our findings. Panel A shows the intervals for each conductance parameter, in which the original trend of the intra-burst ISIs remained unchanged. They can be interpreted as the allowed variability in parameter space in the direction of each particular parameter for which this LP neuron characteristic is conserved. Panel B and C illustrate the underlying data. In each sub-panel the intra-burst ISI is plotted versus the DC current injection for models with the marked percentage change in the value of the *g_CaT_* conductance. For example, the first panel in B is for a model with *g_CaT_* augmented by 0.1% and the last panel in B is a model with *g_CaT_* augmented by 323.97%. The non-integer numbers are due to the logarithmically increasing step size used (see Methods). One can clearly see that the dependence of the intra-burst ISI on the current injection *I_DC_* is initially a linear increase which gets flatter the larger *g_CaT_* becomes and eventually “flips over” at +130.77% (arrow). Similarly, decreasing *g_CaT_* leads to changes in the dependence and eventually a flip at −46.15%(arrow). These limit values of the tolerance are used to create the bar in Figure 10A. The same analysis was carried out for all other maximal conductances resulting in the remaining tolerance bars shown in Figure 10A.

**Figure 11:**
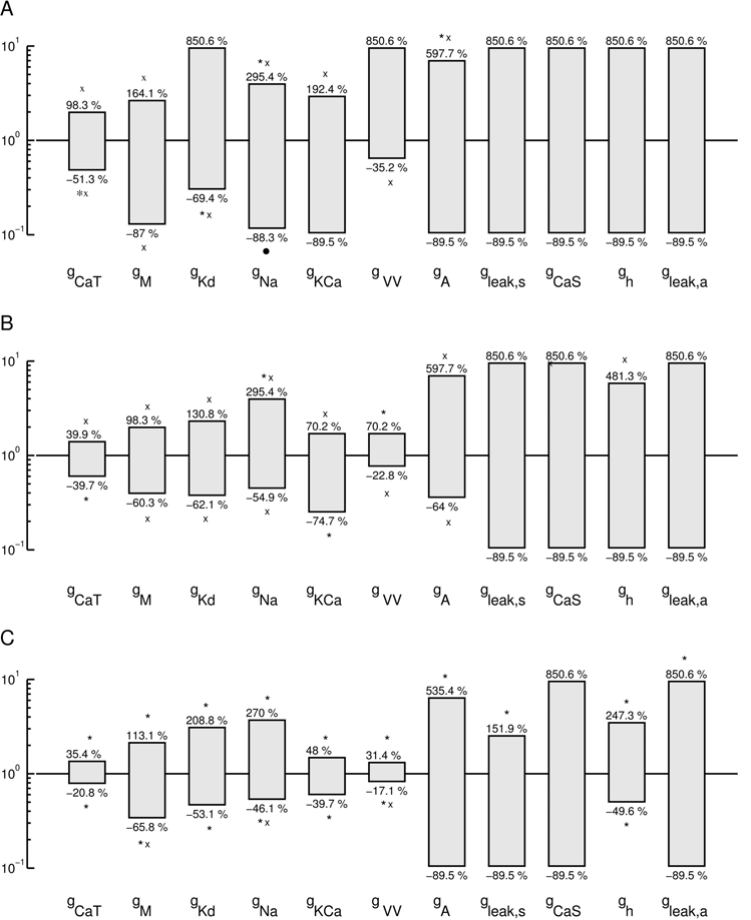
Model tolerance intervals for preserved inter-burst interval trend (A), Burst frequency trend (B), and burst duration trend (C). Burst duration and burst frequency are somewhat more delicate which is reflected in smaller tolerance intervals. Overall the tolerances to parameter changes are, however, remarkably large ranging from a ±17% change (*g_VV_* in B) to −90% and +900% change for many parameters and preserved property trends. (Parameters were sorted in Figures 10 and 11, such that the tolerances are increasing in Figure 10). The marks (‘x’, ‘*’ and bullet) indicate the criterion for the boundary of the tolerance bar as in Figure 10.

A corresponding analysis was used for the inter-burst intervals (Fig. 11A), burst duration (Fig. 11B), and burst frequency (Fig. 11C). Overall, the burst duration and, presumably as a consequence, the burst frequency are the most fragile among the four investigated properties, indicated by smaller tolerance bars for some of the parameters. Unlike the above results using the cost function, the restrictions on *g_KCa_* and *g_A_* are of a similar order of magnitude as those on the other parameters here. This is signaling that the influence of *g_KCa_* and *g_A_* on the model dynamics is comparable in importance to the influence of other parameters.

The conductances are ordered according to their allowed range with respect to the intra-burst ISI results in Figure 10. Clearly, with the exception of *g_KCa_* and *g_VV_* for burst frequency and burst duration, and *g_h_* for burst frequency, the overall order from the most restricted to the least restricted parameters is the same for the four analyzed properties. From the exceptions we conclude that the coupling between compartments *g_VV_* plays an important role for regulating the burst frequency and burst duration, presumably indirectly affecting the former by changing the latter. Similarly *g_KCa_* is more important for burst frequency and burst duration than for the IBI and intra-burst ISI, which confirms its role in burst termination and thus burst duration. It is somewhat surprising that the influence of *g_h_* on the burst frequency is stronger than average but its influence on the IBI and on the burst duration is weaker than average, even though these three properties are strongly inter-dependent. This deserves further investigation in future work.

In summary, the analysis of robustness intervals has revealed that the model is overall much more robust when only the preservation of trends of parameters is required. The possible ranges of parameter values lie between ±17% in the most restrictive cases to virtually unlimited (tested up to 1/10 and 10 times the original value) changes in the least restricted ones. The smaller robustness to changes in *g_CaT_*, *g_M_* for all properties and *g_KCa_* and *g_VV_* for both burst duration and frequency, as well as, *g_h_* for burst frequency alone, indicates that these parameters are the most important for the bursting dynamics of the LP neuron. Parameters like the slow Ca conductance *g_CaS_* and the leak conductance of the axon *g_leak,a_* do not seem to matter for these aspects of the neuron dynamics at all.

### Implications of parameter variability for the estimation procedure

Optimally, there exists a ‘true’ set of parameters, i.e., a set of parameters that reflects the biological reality. This set would correspond to a global minimum of 0 cost and would eventually be found by the estimation procedure. Subsequent repeats of parameter estimation with initial parameter sets close to the ‘true’ set would then lead to the same 0 cost solution. The situation is, however, typically more complicated with many local minima of the cost function. These minima can be well defined (steep) or rather variable with respect to some parameters (shallow). If there is a structural model-misspecification there may not even be a unique global minimum and if it exists it may be far from 0. To assess the situation for our LP model and parameter estimation method we conducted a set of numerical experiments in which a slightly changed parameter set was readjusted using the parameter estimation procedure. We started by changing one parameter, the transient Ca conductance *g_CaT_*, and fitted the model again to the original experimental data. In three trials we adjusted one, two or three of the parameters of the model simultaneously.

**Figure 12:**
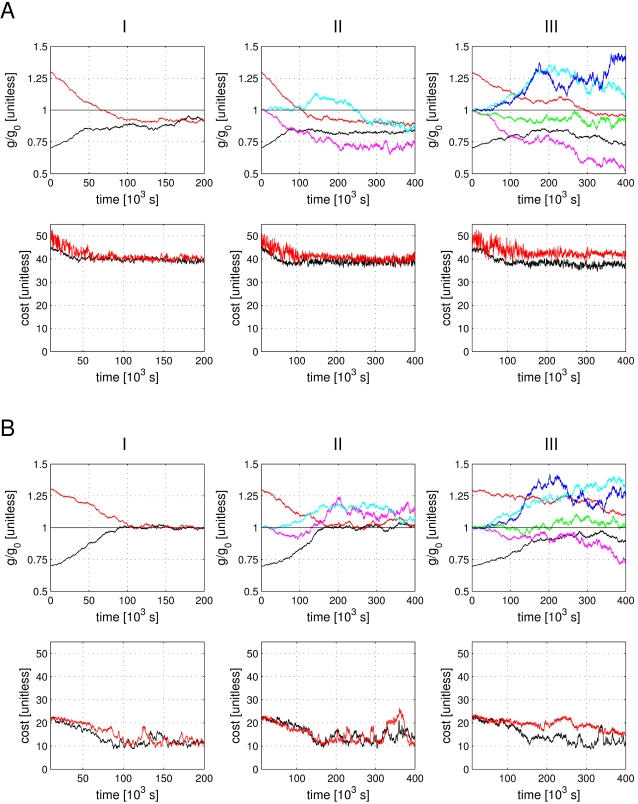
Refitting the model after a 30% change in the *g_CaT_* conductance. A) The model was fitted to the data, after the transient calcium conductance *g_CaT_* was increased (red) or decreased (black) by 30%. I) Top panel, the evolution of *g_CaT_* and bottom panel the cost function during this re-fitting exercise. During the fit, only *g_CaT_* was allowed to change. II) Same as (I) but *g_A_* was released for fitting (magenta and cyan for up and down change of *g_A_* respectively). III) In addition to *g_CaT_* and *g_A_*, *g_KCa_* was allowed to be fitted (blue and green). B) Parameter and cost function evolution as in (A), while the model was fitted to its own output data. The colors and the panel arrangement are the same as in (A).

The results of these refits are shown in Figure 12A. The maximal conductance of the transient calcium current *g_CaT_* was changed by 30% upwards or downwards and the model was fitted again to the experimental data allowing changes in *g_CaT_* only (I), in *g_CaT_* and *g_A_*, (II) and in *g_CaT_*, *g_A_* and *g_KCa_*, (III).

Instead of refitting to the experimental data, the model can alternatively be fitted to its own control output without change in *g_CaT_* (Fig. 12B). While fitting back to the experimental data is closer to the biological reality, using the model's own output has the advantage of revealing more about the sensitivity of the model to parameter changes *per se*. In the case of fitting to the model there is an objectively “correct” solution (the original model) for the estimation procedure. Therefore, it is not necessary to consider trade-offs between different aspects of the cost function that may create many different local minima. The panels in Figure 12B correspond to the panels in A. Like in A, all experiments show a return of the perturbed *g_CaT_* towards its original value.

Not surprisingly, the refit arrives in both cases (A and B) closest to the original parameter value if only the one parameter that was changed is adjusted ((I) in A and B). As more parameters are adjusted concurrently, the solution becomes less unique and a different parameter set (with almost equal cost) is obtained. Note that the cost function is so sensitive that it does not converge to 0 even though the resulting parameter in Figure 12B (I) is equal to the original value for all practical purposes. However, when 3 parameters are adjusted some variation appears in the other parameters while the cost function returns to its original low value ((III) in A and B). This indicates that there is a fairly wide parameter region of viable model solutions with similar performance. The variability in *g_A_* appears somewhat larger than in *g_KCa_* which corresponds to the observation of a greater sensitivity of the model to changes in *g_KCa_* observed directly. It is noteworthy, however, that the upward and downward changes in *g_CaT_* trigger the same direction of compensatory changes in the other conductances in both fits to the data and to the model's own output (compare the blue, cyan, magenta, and green curves in the upper panels of column (III) in A and B) . The results are consistent with the sensitivity analysis insofar that the sensitivity to changes in *g_A_* and *g_KCa_* are on the low end of the spectrum and of a very similar order of magnitude and, therefore, the constraints on the values of *g_A_* and *g_KCa_* are similar and less stringent than for *g_CaT_*.

### Chaoticity

**Figure 13:**
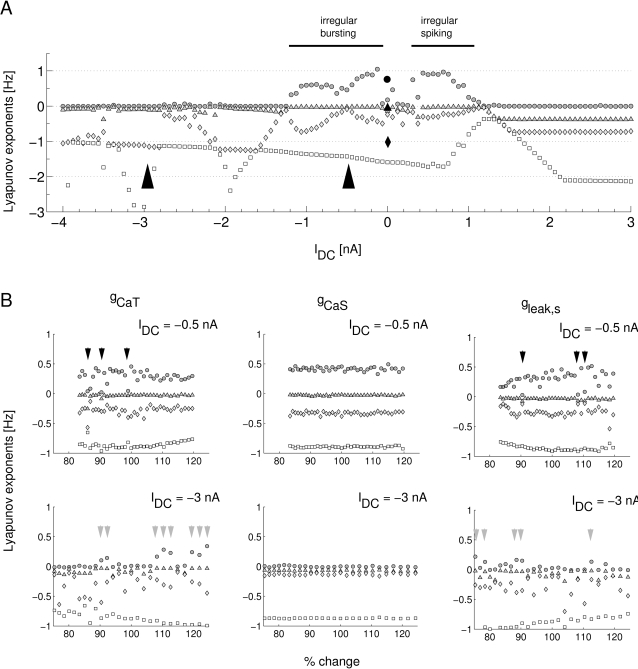
A) The 4 largest Lyapunov exponents of the LP neuron model as functions of the current injection *I_DC_*. The largest exponent is 0 outside the interval [−1.2,1.2]*nA* and in a small region around 0 nA. The positive maximal Lyapunov exponent in the two intervals in between indicates two wide regions of chaoticity. From inspecting the raw data we can identify the dynamics in the two regions to be chaotic bursting for *I_DC_*∈[−1.2,−0.2]*nA* and chaotic spiking for *I_DC_*∈[0.2,1.1]*nA*. The larger, black symbols are the published exponents that were calculated directly from a long recording of an isolated LP neuron at *I_DC_* = 0 [20]. B) Persistence of chaoticity or non-chaoticity for changes in conductance parameters. The upper row shows the 4 largest Lyapunov exponents as a function of the percentual parameter change in *g_CaT_* (left), *g_CaS_* (middle), and *g_leak,s_* (right) at *I_DC_* = −0.5 *nA*. The largest Lyapunov exponent is positive for an overwhelming number of parameters indicating that chaoticity typically persists at *I_DC_* = −0.5 *nA* even if parameters are changed. However, there are some exceptional parameter values (black arrowheads) for which the largest Lyapunov exponent is not unambiguously greater than 0 indicating that the corresponding models are non-chaotic. In the non-chaotic region of *I_DC_* = −3 *nA* the largest Lyapunov exponent is 0 for most parameter values and the models are thus non-chaotic. Again, there are some exceptions of isolated parameter values with different model behavior (grey arrowheads).

It has been reported previously that the membrane potential dynamics of the LP neuron of the lobster have a wide irregular, potentially chaotic, regime [20], [39]. Proving chaoticity by directly analysing experimental data has its limitations, however. Using our model, that has qualitatively the same dynamical characteristics as the biological system, we can bypass the technical problems of estimating Lyapunov exponents from a limited set of noisy experimental data. To test whether our model has one or more chaotic region(s) and how wide it (they) is (are), we calculated the spectrum of global Lyapunov exponents of the model at different hyperpolarization levels. We used a renormalization based method employing the analytical Jacobian of the model equation to calculate the exponents (see Methods for details). Figure 13A shows the 4 largest Lyapunov exponents as a function of the somatic DC current injection *I_DC_*. For *I_DC_*<−1.2 *nA* the largest Lyapunov exponent is 0 indicating that there is no chaotic dynamics present. For *I_DC_* between −1.2 *nA* and 1.1 *nA* the maximal Lyapunov exponent is positive - an indication of chaotic dynamics. Notably, there seem to be two regions of chaos which we may identify as chaotic bursting (*I_DC_*∈[−1.2,−0.2]*nA* and chaotic spiking (*I_DC_*∈[0.2,1.1]*nA*), divided by a narrow window of apparently non-chaotic behavior around 0 nA.

Following our interest in how generic this observation is with respect to changes in parameters we analyzed the changes in the Lyapunov spectrum at two characteristic *I_DC_* levels, *I_DC_* = −0.5 *nA* (well in the chaotic bursting regime with one positive Lyapunov exponent) and *I_DC_* = −3 *nA* (in the regular bursting regime where all non-trivial Lyapunov exponents are negative). Some illustrative examples (for parameters *g_CaT_*, *g_CaS_*, and *g_leak,s_*) are shown in Figure 13B. The chaoticity is a typical property of the neuron models at *I_DC_* = −0.5 *nA* as only few parameter values lead to all-negative Lyapunov exponents (black arrowheads mark candidates). Similarly, non-chaotic dynamics is typical for *I_DC_* = −3 *nA* with only a few examples of positive Lyapunov exponents (grey arrowheads). The situation is similar for all the other conductances (data not shown).

To quantify this observation we classified the models arising from different parameter sets into 3 categories: a) clearly chaotic (the first Lyapunov exponent is positive and at least 5 times larger in amplitude than the Lyapunov exponent closest to 0) b) probably chaotic (the first Lyapunov exponent is positive and at least 2 times larger in amplitude than the Lyapunov exponent closest to 0 but not 5 times larger), c) (probably) non-chaotic (all other cases). For all 11 conductance parameters tested on a range from −20% to +20% parameter change, we observe at *I_DC_* = −0.5 *nA*: 89.1% clearly chaotic, 4.3% probably chaotic, and 6.4% non-chaotic models. At *I_DC_* = −3 *nA* we observed in contrast: 4.1% clearly chaotic, 9.1% probably chaotic, and 86.8% non-chaotic models. While “counting results” of this type have to be taken with a grain of salt (see [47] for an in-depth discussion), we would like to argue that this result indicates that at *I_DC_* = −0.5 *nA* chaoticity is a typical trait of the models which are close to our model in parameter space. By the same token, models similar to ours are typically non-chaotic at *I_DC_* = −3 *nA*.

When comparing the different parameters, we again notice that the value of *g_CaT_* has a much larger impact on the dynamics than the value of the slow Ca conductance *g_CaS_* which seems to have no impact on the chaoticity of the neuron model at all (Fig. 13B left and middle). This is consistent with the ultra-low sensitivity of the model to the value of *g_CaS_* with respect to the cost function and the measurement of general trends.

When comparing the range of ±20% change in parameters to our sensitivity results above we notice that changes of this magnitude are mid-way between the high sensitivity with respect to the cost function and the low sensitivity for the general trends. This matches the observation that only few models differ with respect to chaoticity within this range while the overall majority of models are consistent.

It is noteworthy that the parameters that led to non-chaotic behavior at *I_DC_* = −0.5 *nA* as well as the parameters leading to chaos at *I_DC_* = −3 *nA* do not seem to cluster but are interspersed with parameter sets that generate the ‘typical behavior’. This indicates that the parameter region of models with a given property (like chaoticity) is highly complex. There always seem to be close-by parameters that lead to a different model, confirming similar observations in a recent related work [48].

### Model predictions

Models can allow predicting future experimental results and the design of new experiments and thus provide insights that are otherwise impossible. In the case of our conductance based model of an identified cell, the structural similarity of the model to the biological system in how ionic currents combine to generate a membrane potential waveform should allow us to predict the effects of manipulations of individual conductances.

A well-known phenomenon in the pyloric circuit is, that slow oscillations of neurons persist even when action potential generation is completely blocked with TTX [49]. To simulate the action of TTX we set the maximal conductance *g_Na_* of our model neuron to 0 and tested the usual range of DC steps (Fig. 5B). We observed dynamics that were similar to the observation in the biological preparation (cf. [49], Fig. 1D), even though the latter was obtained in the intact circuit. In particular, the slow waveform of the regular and irregular bursting patterns with additional small oscillations within long bursts remains intact independently of the blockage of spiking. This is a clear indication that irregularities of the burst duration do not depend on an interaction between spike generation currents and slower burst generating currents.

**Figure 14:**
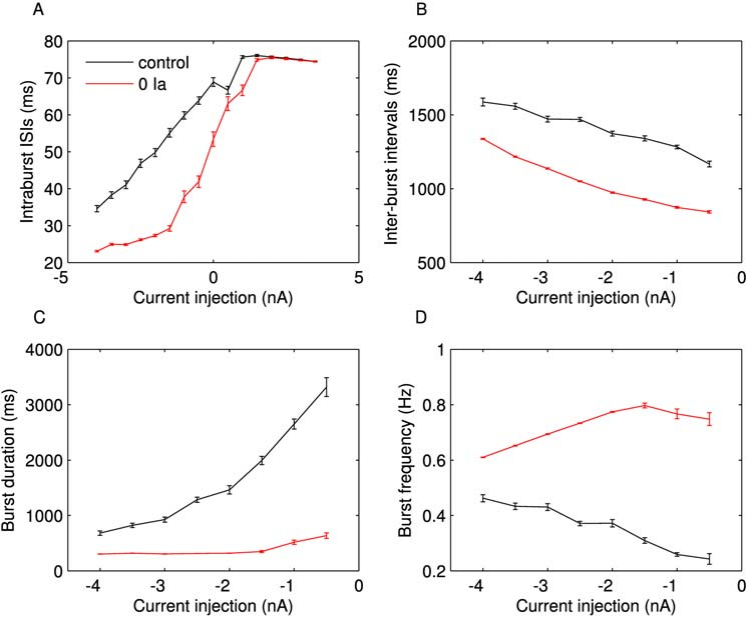
Predictions for the effect of selectively blocking the A current – *I_A_* (red lines) compared to the control condition (black lines). The expectation is for unchanged trends in the intra-burst ISIs, the IBIs, and the burst duration (A–C) whereas the burst frequency is expected to change in the hyperpolarized regime from decreasing with less hyperpolarization to increasing (D). In absolute values we would anticipate reductions in intra-burst ISIs, the IBIs, and the burst duration (A–C) and an increase in burst frequency (D). In light of our observations of consistency of neuron properties in biological LP neurons we would expect more predictive value for the prediction on the trends than for the prediction on absolute values.

In a similar way, we can block any of the currents in the model. An attractive candidate, due the availability of relatively specific drugs, is the *I_A_* current. In Figure 5C, the maximal conductance *g_A_* of this current is set to 0. Unlike in the previous “numerical experiment” of blocking *I_Na_*, blocking *I_A_* has less disruptive effects on the neuron dynamics. With the naked eye the overall activity patterns appear almost unchanged with tendency to slightly less irregularities in bursts. When analyzed with the statistical measures (Fig. 14) we see clear trends of smaller intra-burst ISIs, smaller IBIs and smaller burst durations when *I_A_* is blocked. The two latter effects result in a higher burst frequency. Interestingly, while the trends of the first three quantities remain similar to the original model, the burst frequency changes its trend and increases with less depolarization for *g_A_* = 0 while it decreases in control conditions. In recent work the effect of blocking *I_A_* with 4-aminopyridine (4 AP) in the intact circuit was investigated [10]. While direct comparisons of circuit results and results for an isolated cell are difficult, we do note that in both cases the burst frequency of the LP neuron increases. For the burst shape measure B as defined in [10], results are less clear. Its change in the experiments was not significant and its change in the model is of similar magnitude but in a different direction (at *I_DC_* = −5 *nA* we observed B = 0.439 in control and B = 0.264 with *g_A_* = 0). Overall these predictions seem to be consistent with existing experimental knowledge. A more definitive answer will need a more careful study of the action of ion channel blockers in isolated LP neurons.

## Discussion

With the progress in our understanding of neuronal systems and the development of new and more quantitative experiments it is becoming possible to ask not only whether variability in electrophysiological properties of neurons exists, but also how large it is and what it means for neuronal function. The most direct methods for studying this question involve massive experimentation with large numbers of animals. The use of computer modeling may be a way to aid experiments and augment the obtained results. Here we used an approach for automated parameter estimation in data driven models and investigated the variability of neuron properties and underlying parameters with the resulting models.

Clearly, a prerequisite for such an effort is the existence of accurate models that replicate the behavior of neurons adequately. Even though phenomenological, in a strict sense, conductance based neuron models are structurally close enough to the actual neuron membrane processes to raise the expectation that they may provide such models and allow predictions beyond a mere mimicry of the observed membrane potential dynamics. Nevertheless, contrary to the first impression of a rather mature field given the large number of available conductance based models of various cells, the computational neuroscience at this level - the description of typical activity patterns of identified cells and the prediction of activity changes in response to perturbations - seems to still be in its early stages. For instance, one may reasonably expect that a well-tuned and accurate conductance based model would predict the changes in neuron activity if one of the ionic currents was blocked. Many existing models, however, seem to struggle to perform such a task. Even though a few models have been more successful [50]–[54] these often are either extremely detailed [52]–[55], or the result of a decade long production of models involving several generations of researchers and their hand-tuning efforts [50], [51]. Both seem impractical for future data-driven models in computational neuroscience. Using data fitting or automatic parameters estimation techniques for building data-driven models of elements of the nervous system, in particular neurons, is becoming more and more popular [45], [56]–[62]. The specific requirements of these efforts differ quite substantially, however, and many challenging technical problems remain. In particular, if the experimental knowledge about morphology and channel kinetics exists only partially, there still seems to be no simple standard procedure to build a predictive H-H model to a given set of observations. The main open problems of automated parameter estimation for HH models lie in three areas - what data to use (data set), how to measure the similarity of model output and observed neuron activity (cost function), and what algorithm to use for optimization (algorithm). In making these difficult choices it may be very important to take into account the function of the neuron under consideration. Our results suggest that in some cases, like for the isolated LP neurons considered here, the properties that define an identified cell or a certain class of neurons can be less obvious than the spike shape, burst shape, absolute spiking or bursting frequencies. Some of the most salient properties of the observed neuron dynamics may be the characteristic changes of a property in response to certain types of stimulations (bifurcations) rather than their values during any given stationary activity (the attractor(s)). In the study presented here, the most salient property was the characteristic transition from bursting to spiking depending on hyperpolarization and the ensuing typical change in burst duration and inter-burst interval. In other neurons it may be certain response characteristics to PSPs and other response properties. Our choices for the particular method employed here were informed by the direct comparative analysis of data from isolated LP neurons as well as the existing literature on the lobster STG. We observed that characteristic trends of change in neuron properties in response to different levels of de- and hyperpolarization appear to be more consistent then absolute values for those properties. This is an interesting observation in its own right and after some thought seems very plausible. After all, LP neurons have to function within a network in which they constantly receive (dominantly inhibitory and slowly modulating) input. A consistent response to such inputs may be much more relevant than consistent dynamics in isolation.

For our model building procedure this insight prompted us to use a data set containing a wide range of dynamics at different levels of current injection. This also seemed appropriate from a dynamical systems point of view because it is *a priori* not clear how much the dynamics in one region of state space and under one stimulus condition can reveal about other regions and for other stimuli. This is particularly true if two conditions are separated by a bifurcation in the system's dynamics. For the model of the LP neuron developed here, using a wide range of data and an appropriate cost function led to a faithful reproduction of the observed trends in neuron properties without the necessity of explicitly referring to these properties during the model development.

Our choice for the cost function was driven by the common consensus that the neurons in the STG mainly communicate based on bursts of spikes even though there have been suggestions for a more fine-grained picture where the timing of spikes within bursts becomes relevant [63]. In support of our choice we observed that the spiking patterns within bursts are reasonably controlled in spite of the more coarse-grained cost function.

The third choice, the optimization algorithm, seems less important than one might naively expect. Comparative studies have shown that both simulated annealing and genetic algorithms typically perform well. Our choice of simulated annealing over genetic algorithms was inspired by ideas for potential online fitting procedures.

With the choices made, our automatic parameter estimation procedure for a model of the LP neuron of the lobster, resulted in a successful model with some predictive power. At the same time there still remains some room for improvement. Nevertheless, we have collected more evidence that automatic parameter estimation does hold the promise of moving the focus from conceptual models to experiment-based, and therefore more powerful, data-driven models in the future.

The analysis of the experimental data and the model revealed additional insights into the variability of LP neurons. By inspection of the data at many different levels of current injections we observed that the variability across neurons in a given condition (current injection) may merely reflect a shift of the dynamical patterns of activity across conditions (current injections). An example of this effect is the wide variability found between the individual LP neurons at *I_DC_* = 0 *nA* current injection. Activity patterns at 0 nA injection in one neuron are often more similar to patterns at −0.5 *nA* in other neurons than at the corresponding 0 nA injection level. This “shift effect” could be explained by different leak currents due to additional leaks caused by the electrode insertion into the neuron membrane. A similar effect has previously been observed in the leech heartbeat network. In this case, the HN3,4 neurons were thought to be non-bursting when recorded intra-cellularly [64]. However, when recorded extra-cellularly, i.e., without any damage to the membrane, they turned out to be bursting cells [65]. Translated to the case at hand this may signify that the LP neurons may actually be much more similar than it would appear from intra-cellular recordings of the control behavior. The protocol of several different current injections used here helps detect such an observation bias because it allows the detection of trends rather than individual points.

Adding to a number of previous studies [5], [23], [66]–[68] we tried to link the observed variability in dynamics to the properties of the neurons (parameters of the neuron model) and observed that the notion of parameter sensitivity depended strongly on the criteria for “essentially equivalent model dynamics” (see also the discussion in [4], [47]). Clearly, if the dynamics are to be exactly reproduced in terms of the components of the cost function (normalized SDF, total SDF, and moving average of the membrane potential) the restrictions on parameters are more stringent than if only the reproduction of a trend in a dynamical property (intra-burst ISI, IBI, burst duration, or burst frequency) is required. The result of our analysis lies in the extent of this difference. In the former case, some parameters are restricted to a minute range of down to ±0.1% whereas in the latter case a ±15% change did not seem to matter for any of the parameters and many parameters could be changed ten-fold or more without an effect. While surprising at first, these extremely wide ranges of meaningful parameter values match recent observations in biological systems in general [69], [70].

In this work we only analyzed the sensitivity of the model to changes of one single parameter at a time. There are now indications that some currents may be coregulated [71], [72]. Coregulation raises the possibility that changing one current at a time may seem to have catastrophic effects while, at the same time, adequately compensated changes of multiple current may have well-defined (and desired) effects. Without an extensive investigation we can also not exclude that the opposite is true and the observed co-regulation is designed to enhance the effect of changes in the currents. However, theoretical examination of the parameter space using models may be a powerful tool in finding the control parameters used to regulate neurons. For example [33] found combinations of parameters that when regulated preserve the activity of a model neuron and its characteristic dynamical profile.

As another example for indirect neuron analysis using our model, we revisited the question of the nature of the observed irregular behavior of LP neurons over a wide dynamical range of conditions [22], [39]. We were able to show that in the model this can be explained as properly chaotic behavior (indicated by positive Lyapunov exponents, Fig. 13) arising from the interaction of slow and fast ionic conductances and a slow first order Ca dynamics. This confirms earlier direct observations on LP membrane potential time series [20]. Interestingly we found two broad chaotic regimes which are separated by a narrow region of more regular activity. We tentatively identified the two regions of chaotic behavior as chaotic bursting and chaotic spiking.

It has been suggested that the irregular (chaotic) dynamics in LP neurons is connected to the Ca dynamics [29], [73], [74]. Our results are consistent with this hypothesis but the involvement of internal Ca stores as suggested in [29] does not appear to be necessary. We also did not need to invoke stochasticity as suggested in [75].

When analyzing the robustness of the observed chaoticity against parameter changes for two illustrative current injection levels, we observed that the typical behavior of chaoticity or non-chaoticity is preserved for most parameter sets but interspersed are sets leading to different behavior. This indicates a highly complex parameter space landscape with very similar and very different models in close neighbourhood as has been recently observed independently for different classes of neurons [48].

The analysis of the impact of changes to individual parameters on the observation of chaoticity is consistent with our observations with respect to the other properties investigated (similarity to the original model measured by the cost function, similarity to the original model measured by trend changes in the dependence of ISI, IBI, etc. on current injection). Parameters that did not seem to matter much for the former (e.g., *g_CaS_*) also did not matter for the latter and those that were important for one parameter (e.g., *g_CaT_*) were always important for both.

We have argued before that a well-adjusted HH model may predict the effect of blocking individual ionic currents. Here we made two such predictions. If the sodium currents are blocked we preditced a dynamics of “naked bursting” in which the slow burst waveforms remain almost unaltered but do not lead to spikes (Fig. 5B). For blockage of *I_A_* we predicted almost undisturbed bursting and spiking activity (Fig. 5C) with slight changes in bursting frequency and duration. Both predictions are consistent with available related data ([49], in case of *I_Na_*) and preliminary results (R.L. unpublished). The specific predictions remain to be tested. A verification of our predictions, even if only partially, would be another piece of evidence that data driven modeling similar to ours can be sucessful.

The dynamical properties of neurons are determind by their structure and current composition and are crucial for their function. Therefore, a combined approach, such as is presented here, that uses data driven modeling in the context of functioning neurons can promote better understanding of the function of both neurons and neuronal systems. One advantage of using automated methods is the possibility of gaining insight into possible mechanisms of parameter adjustment in the biological system. It is clear that some genotype variability in addition to changes during the life of the animal exists. If the control parameters that need to be maintained are known, it will be possible to search for the biological mechanisms that generate the control. The approach promoted in this work has the potential of revealing the most salient control parameters as candidates for such an endeavor. Combined with experiments which are driven by the theoretical predictions it can be a powerful tool in the analysis of neuronal system function.

## Materials and Methods

### Electrophysiology

We used pacific spiny lobsters *Panulirus interruptus* supplied by local fishermen. After cooling the lobsters in ice for anesthetization, the STG was removed along with the commissural and esophageal ganglia as described previously [7], [76]. The preparation was continuously perfused with saline at room temperature. Individual neurons were identified by correlating intracellular recordings with corresponding extracellular recordings from identified nerves. The experiments were performed in standard *Panulirus* saline, which had the following composition (in mmol/l): 479.1 *NaCl*, 12.7 *KCl*, 13.7 *CaCl*_2_, 10.0 *MgSO*_4_, 3.9 *Na*_2_ *SO*_4_, 5.0 HEPES, and 5.0 TES, pH 7.4. We isolated LP neurons of the pyloric system with photoablation [77] and chemical inactivation of synapses with 10^5^ M picrotoxin (PTX, Sigma) as described earlier [78]–[80]. The intracellular recordings were made with 8–10 MΩ glass electrodes filled with 3 M *KCl* using intracellular amplifiers (AM systems Inc., WA). All data were stored on Axoscope (Molecular Devices Inc., CA) for later analysis. We then obtained long recordings of the membrane potential of the isolated neuron subject to DC current injections applied through a second electrode inserted into the cell soma.

### Model development

We built a conductance based model of the lobster (*Panulirus interruptus*) lateral pyloric (LP) cell using a set of parameter estimation (fitting) techniques. The initial model and the model parameters before parameter estimation were derived from previous experimental and modeling results. In particular, for the transient calcium current, *I_CaT_*, the slow calcium current, *I_CaS_*, and h current, *I_h_* we refitted standard H-H type equations to LP voltage clamp data [18], [19], [23], taking into account the advances in data fitting [81] made since the initial efforts of model building were undertaken [24]. For the calcium induced potassium current *I_KCa_* and the A current *I_A_* we used the existing fits to LP data of the crab (*Cancer borealis*) [24]. The fast sodium, *I_Na_* and delayed rectifier, *I_Kd_*, currents have not been experimentally jointly characterized in the LP neuron of either lobster or crab due to space clamp issues with the Na current. The Kd current was recently measured in *Panulirus* [82]. Using this data without corresponding data for the Na current unfortunately would make it close to impossible to achieve appropriate spiking activity in the initial model due to the intricate balance between Na and Kd currents in spike generation. To ensure stable and appropriate spike generation we resorted to standard equations [83] for Na and Kd currents and did not adjust them during the fitting procedure. The spike rate adaptation current *I_M_* was introduced based on the observation of spike rate adaptation in all of our data sets. Unlike the activation and inactivation parameters of other currents, all parameters of this current were subject to a full fit. The calcium dynamics in the cell was described by first order dynamics and without spatial resolution.

We grouped the currents into two compartments, *I_CaT_*, *I_CaS_*, *I_KCa_*, *I_A_* and *I_h_* in a compartment we denote by ‘soma’ and the remaining currents, *I_Na_*, *I_Kd_* and *I_M_* in a compartment denoted ‘axon’. This division implies a functional distribution rather than a strict physical one, but is supported by experimental results that demonstrated substantial distribution of Calcium currents close to the soma [84], [85]. The strong attenuation of spikes observed in the soma, on the other hand, indicates that the spike generating currents are located electrically far from it. Both soma and axon have a separate leakage current *I_leak,s_* and *I_leak,a_* respectively. The unknown parameters of membrane capacitance of soma and axon compartments and in particular the coupling between compartments were initially adjusted by hand to obtain reasonable activity patterns in the initial model.

### Model equations

The membrane potential of axon and soma compartment, *V_axon_* and *V_soma_* respectively, are described by
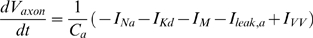
(9)

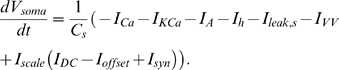
(10)


*I_offset_* = 2 *nA* stems from the residual coupling at the end of the parameter estimation procedure which introduces a current bias due to differences in baseline voltage of data and model (on top of *V_shift_*). *I_syn_* denotes the total incoming synaptic current. All ionic currents except for the ones mentioned explicitly below are given by
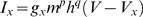
(11)where is the maximal conductance of the current, *V* is the membrane potential of either the axon compartment (*I_Na_*, *I_Kd_*, and *I_M_*) or the soma compartment (all other currents), and *V_x_* is the reversal potential of the current. The activation and inactivation variables are governed by
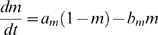
(12)

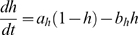
(13)for *I_Na_* and *I_Kd_*, and
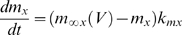
(14)

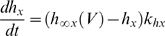
(15)for the other currents, denoted by *x* = *CaT*, *CaS*, *KCa*, *A*, *h*, and *M*. The calcium current is formulated in the Goldman-Hodgkin-Katz (GHK) formalism
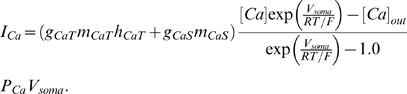
(16)


The *I_A_* current has two different components of the inactivation variable,
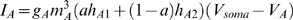
(17)

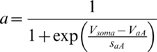
(18)


The leak currents are given by
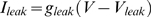
(19)with *g_leak_* = *g_leak,s_*, *g_leak,a_* and *V* = *V_soma_*, *V_axon_* for the soma and axon leak currents respectively. The calcium concentration is described by a first order kinetic equation,
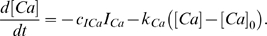
(20)


Finally the coupling between compartments is ohmic, *i.e.*,
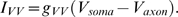
(21)


The activation and inactivation functions *a_m_*, *b_m_*, *a_h_*, *b_h_*, and *m*_∞_, *h*_∞_, *k_m_*, *k_h_* are described in Table 5. The parameters that were typically not subject to adjustments are summarized in Table 4. One parameter set of adjusted parameters is shown in Table 3.

**Table 5:** Activation and inactivation functions for *I_Na_*, *I_Kd_*, *I_Ca_*, *I_KCa_*, *I_A_*, *I_h_*, and *I_M_*.

	p	q	*a_m_*	*b_m_*	*a_h_*	*b_h_*
*I_N_*	3	1	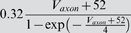	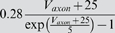	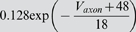	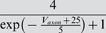
*I_Kd_*	4	0	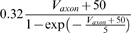	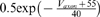		
	p	q	*m*_∞_	*h*_∞_	*k_m_*	*k_h_*
*I_CaT_*	1	1	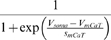	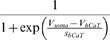	*k_mCaT_*	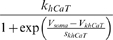
*I_CaS_*	1	0	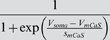		*k_mCaS_*	
*I_KCa_*	1	1	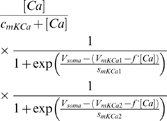	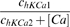	*k_mKCa_*	*k_hKCa_*
*I_A_*	3	1,1	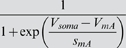	*h_A_*_1_ & *h_A_*_2_: 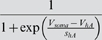	*k_mA_*	*h_A_*_1_ : *k_hA_*_1_ *h_A_*_2_ : 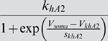
*I_h_*	1	0	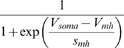		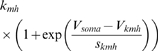	
*I_M_*	1	0	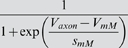		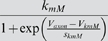	

The activation and inactivation curves of currents were taken from the literature or fitted to data from the literature, in particular, *I_Na_* and *I_Kd_* from [52], *I_KCa_*, *I_A_* directly from [24], *I_h_* is our own fit to voltage clamp data in [24] using the same functional form as in [24], and *I_Ca_* is our fit to voltage clamp data in [23], using the standard GHK formalism.

*I_M_* is not based on direct experimental observation and implements a generic M type spike rate adaptation current. All its parameters were subject to fitting.

### Detailed cost function

In automated fitting (parameter estimation) algorithms the quality of a set of parameters in describing the target data is measured by a so-called cost function. The cost function in our procedure was the weighted sum of three cost measures computed from the model output and the data (or the output of two different models). The main contribution is the Euclidean distance between the normalized spike density functions (SDFs), [46], of the model and the data, denoted by *s*_model_(*t*) and *s*_data_(*t*):
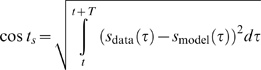
(1)where T denotes the width of the time window of comparison between model and data. The normalized SDFs are given by
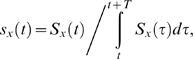
(2)where x stands for ‘model’ and ‘data’ respectively and the total SDF is defined as
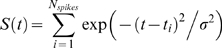
(3)


The variables t_i_ denote the times when the ith spike occurs and the standard deviation of the Gaussian curves is *σ* = 100 *ms*. This choice for *σ* ensures that individual spikes are not resolved individually but rather contribute to a global burst shape. At the same time, typical bursts are still well-separated in the normalized SDF.

**Figure 15:**
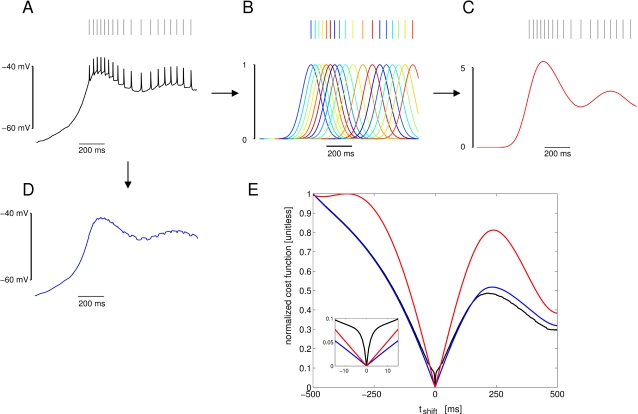
Components of the cost function and how they are calculated. A) An Example of a membrane potential trace from the LP model with gray bars indicating timing of detected spikes. To obtain the corresponding spike density function (SDF), a Gaussian of fixed width *σ* = 100 *ms* and amplitude 1 is added at each time of spike occurrence (B). The colors in (B) illustrate which spike is represented by which Gaussian. The summation of all curves results in the SDF shown in (C). The moving average of the membrane potential is formed by averaging its 100 previous time steps, in this example of about 0.4 ms each (D). E) Illustration of the typical contribution of the cost function components. Here, the time series was time-shifted and compared to an non-shifted version of itself at varying time shifts (x axis; the inset is an enlargement of the region around 0). The dependece of a direct membrane potential cost function on time shift is shown in black (based on the Euclidean distance between all points of the membrane potential and the shifted membrane potential). The dependence of the SDF cost function (the Euclidean distance between all points of the two SDFs) on time shift is shown in red, and the cost for the moving average (the distance between the averaged membrane potentials at varying time shifts) is shown in blue. Note, how both the SDF and moving average widen the “valley” around the minimum where the data is not shifted and thus has 0 distance. It is, therefore, easier to find the minimum with a search algorithm if these types of cost functions are used rather than a direct membrane potential difference. While in this respect similar, the SDF-based distance differs from the distance based on the moving average in that it is sensitive to the existence of spikes. A “naked burst” like shown in Figure 5B for blocked *I_Na_* would, when compared to the control data, lead to small cost for the moving average, but immense cost for the SDF.

Figure 15 illustrates the construction of the cost function graphically. For the first cost function component, spikes are detected in the observed membrane potential data (Fig. 15A) and for each spike a Gaussian bell curve is placed at the time it occurred (Fig. 15B). These Gaussians are then summed up to form the SDF (Fig. 15C). We normalize the SDF by dividing through the overall area (such that the overall area becomes one) and compare the resulting normalized SDFs at each point in time. By doing so, we get a measure of similarity of the pattern of spiking without considering the overall spike rate.

The second component of the cost function is the difference between the integrals of the SDFs over the width T of the comparison time window,
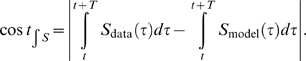
(4)


This measures the (dis)similarities of overall spike frequency rather than the pattern of spiking as the integral of the SDF is proportional to the total number of spikes that were observed.

The normalized SDF and the integral over the SDF were used separately in these two components of the cost function to allow weighting the importance of spike patterning and overall spiking frequency independently. These two first components of the cost function solely depend on spike times and can be viewed as a spike-time dependent cost function with independent weights for spike patterning and overall spike frequency.

The last cost function component is the Euclidean distance of a moving average of the membrane potentials,
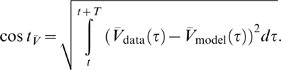
(5)where the moving average *V̅*(*t*) is the average of the membrane potential over the last 100 time steps of numerical integration up to t (Fig. 15D). This component of the cost function measures the slow waveform of the membrane potential and is not particularly sensitive to spikes.

The three components were combined in a linear sum, cos *t* = *k*_s_ cos *t*_s_+*k*_∫*S*_ cos *t*_∫*S*_+*k_V̅_* cos *t_V_*_macr;_ with weights k_x_ that were chosen empirically such that the first component (cos *t_s_*) accounts for about 90% of the cost, the second (cos *t*_∫*S*_) approximately 9%, and the last component (cos *t_V̅_*) the remaining 1% (see the main text for a further discussion of this choice). This compound cost function was measured simultaneously in several sliding time windows of length T and the results were added linearly to determine the total cost.

Figure 15E illustrates how the different components of the cost function perform in comparing a typical burst of the model to a time shifted version of this burst. In this mock example the moving average of the membrane potential seems to provide very similar information as the spike density function. Note, however, that the moving average is not spike-sensitive and would look almost identical if the burst was just a membrane potential plateau without spikes (see, e.g., Fig. 5B) whereas the SDF would change dramatically in this situation.

### Protocol for parameter changes

The parameters of the model fall into two qualitatively different categories, which we designated as additive and multiplicative parameters. Additive parameters are mainly potentials (i.e., expressed in mV units) and meaningful changes in these parameters are additions/subtractions of small steps (in terms of absolute values in mV), allowing transitions through 0 if they occur. Multiplicative parameters comprise all other parameters we considered, most prominently maximal conductances of currents. Changes in these parameters are best expressed in terms of percentage change, i.e., by multiplication by a factor close to 1. These parameters cannot cross 0 during the fit.

All parameters were hard-limited to an interval of [*p*_0_−50 *mV*, *p*_0_+50 *mV*] (additive parameters) and ⌊*p*_0_·0.1, *p*_0_·10⌋ (multiplicative parameters), where *p*_0_ denotes the initial parameter value. Due to the rather generous size of the allowed intervals we did not observe parameters approaching their hard limits in our final runs.

During the parameter estimation, the size of parameter changes was adjusted dynamically according to the response of the current model to such changes using the following algorithm: If the suggested parameter changes in an annealing step led to a “lateral cost” between the perturbed and unperturbed model that exceeded a given cost maximum, the step size of all parameters, that had been subject to suggested changes, is reduced by a constant factor. If this lateral cost (the size of the change of model behavior in response to the suggested parameter changes) is too small, step sizes are increased. The “target cost” between models was slowly reduced over the duration of the parameter estimation procedure.

In the experiments designed to assess model robustness and sensitivity, the conductances were changed in a range from 0.1 to 10 times their original value, corresponding to the hard limits used in the original parameter estimation. Test steps in parameters for this part of the numerical work were chosen to start with *p*′ = exp(±Δ*p*_0_)*p* with Δ*p*_0_ = 0.001 and continue as *p*′ = exp(±Δ*p_n_*)*p*, with Δ*p_n_* = 1.05*^n^*Δ*p*_0_. Using this “double logarithmic” scale allowed us to characterize parameters both with extremely high and very low sensitivity.

### Low frequency noise

For realistic comparison of the model with the, by necessity, noisy data of the electrophysiological recordings, we added weak synaptic input from 10 Poisson neurons (*V_rest_* = 60 mV, *V_spike_* = 50 mV, *λ* = 60 Hz, 2 ms spike width, 8 ms refractory period) with balanced excitation and inhibition to the LP neuron model. Synapses were described by a standard model,
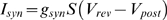
(6)

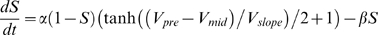
(7)with *g_syn_* = 10 *nS* and probability 0.5 for excitation (*V_rev_* = 20 *mV*) or inhibition (*V_rev_* = −80 *mV*). *α* = 0.1 *ms* ^−1^, *β* = 0.02 *ms* ^−1^, *V_mid_* = −20 *mV*, and *V_slope_* = 10 *mV*. *V_pre_* and *V_post_* denote the pre- and postsynaptic membrane potential respectively. We chose synaptic noise of this type over high frequency Gaussian noise because it has more appropriate timescales for simulating the effects of potential residual interactions of neurons in the experiments.

### Lyapunov Exponents

The Lyapunov exponents were calculated using a renormalization-based method [86], [87] employing the analytical Jacobian of the model equations. The Jacobian was calculated using Maple (Waterloo Maple Inc. (Maplesoft), Waterloo, Ontario). For each DC injection level a trajectory of *T* = 500000 *ms* was calculated for the model and a basis in tangent space which was then re-orthonormalized every 5 *ms* using a Gram-Schmidt orthonormalization procedure. Lyapunov exponents were calculated from the set of the N = 100000 renormalization constants 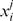, *i* = 1,…,*N*, *j* = 1,…,16, as
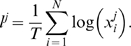
(8)


### ISI return maps

Inter-spike interval return maps were generated by recording the occurrence of each pair (*ISI_n_*, *ISI_n_*_+1_) in a 2D histogram of bin size 1 by 1 ms, and smoothing this histogram by convolution with a Gaussian with standard deviation 5 bins in each x and y directions. The resulting “ISI return map density function” was visualized in a “heat” color map with cold colors for low values and warm colors for high values.

### Statistics

Error bars used in plots are SEM unless stated otherwise. For comparing model parameters to LP parameters we used Student's t-test with 0.05 significance level. For the correlation statistics we used Pearson correlation. All tests were done in Matlab (MathWorks - Inc., Natick, Mass.).
