# Correction: Probing the Dynamics of Identified Neurons with a Data-Driven Modeling Approach

**DOI:** 10.1371/annotation/08b55d6b-7872-40a1-9988-b4bd1c849b8a

**Published:** 2012-10-17

**Authors:** Thomas Nowotny, Rafael Levi, Allen I. Selverston

There are two typographical errors in the equations in Table 5 that make it impossible to reproduce the model:

1. In the I_Kd_ row, third column (titled "a_m_"), the leading constant should have been 0.032 rather than 0.32.

The correct formula is: (0.032 * ((V + 50.0) / (1.0 - exp(-(V + 50.0) / 5.0))))

2. In the I_h_ row, in the 5th column (titled "a_h_") the multiplication "x" should have been a division "/" (or the whole been a fraction).

The correct formula is: (k_mH_ / (1.0 + exp((V - V_kmH_) /S_kmH_))) 

